# The trade-off between fix rate and tracking duration on estimates of home range size and habitat selection for small vertebrates

**DOI:** 10.1371/journal.pone.0219357

**Published:** 2019-07-10

**Authors:** Lucy J. Mitchell, Piran C. L. White, Kathryn E. Arnold

**Affiliations:** Department of Environment and Geography, Wentworth Way, University of York, Heslington, York, United Kingdom; CSIRO Townsville Australian Tropical Sciences and Innovation Precinct, AUSTRALIA

## Abstract

Despite advances in technology, there are still constraints on the use of some tracking devices for small species when gathering high temporal and spatial resolution data on movement and resource use. For small species, weight limits imposed on GPS loggers and the consequent impacts on battery life, restrict the volume of data that can be collected. Research on home range and habitat selection for these species should therefore incorporate a consideration of how different sampling parameters and methods may affect the structure of the data and the conclusions drawn. However, factors such as these are seldom explicitly considered. We applied two commonly-used methods of home range estimation, Movement-based Kernel Density Estimation (MKDE) and Kernel Density Estimation (KDE) to investigate the influence of fix rate, tracking duration and method on home range size and habitat selection, using GPS tracking data collected at two different fix rates from a small, aerially-insectivorous bird, the European nightjar *Caprimulgus europaeus*. Effects of tracking parameters varied with home range estimation method. Fix rate and tracking duration most strongly explained change in MKDE and KDE home range size respectively. Total number of fixes and tracking duration had the strongest impact on habitat selection. High between- and within-individual variation strongly influenced outcomes and was most evident when exploring the effects of varying tracking duration. To reduce skew and bias in home range size estimation and especially habitat selection caused by individual variation and estimation method, we recommend tracking animals for the longest period possible even if this results in a reduced fix rate. If accurate movement properties, (e.g. trajectory length and turning angle) and biologically-representative movement occurrence ranges are more important, then a higher fix rate should be used, but priority habitats can still be identified with an infrequent sampling strategy.

## Introduction

Effective species conservation management requires detailed knowledge of a species’ ecology [1], including but not limited to, an understanding of movement and resource use to make appropriate management decisions that will help conserve populations [2,3]. For certain groups of species, such as small, nocturnal or range-limited species, gathering this information can be logistically challenging. As such, researchers are mostly reliant on indirect observation methods, such as animal-attached devices, including Very High Frequency (VHF) tags, geolocators and Global Positioning System (GPS) units [4] to make an assessment of what habitats are being used [5–7].

The rapid advancement of tracking technology has allowed us to remotely gather information on a wide variety of species [8,9], which can be used to answer questions about how the animal interacts with the landscape, how it moves in relation to habitat type and structure [5,10], its territoriality and interactions with conspecifics [11], and its foraging strategy [12]. GPS units in particular are associated with the ability to collect data from more locations, including previously inaccessible areas, at a higher level of accuracy than before [13,14]. Researchers attaching GPS devices are reliant on the assumption that the data are producing accurate, consistent representations of the animal’s spatial and temporal activities [15–17]. However, studies have shown that movements and habitat usage patterns may be represented differently at different temporal and spatial scales [18,19] and use different methods of home range and habitat selection estimation [20–23], which may or may not take into account the autocorrelation structure of the data [24]. Previous studies addressing these issues recommend obtaining data from multiple temporal and spatial scales for comparison [19,25], and have focused on large mammals [25,26] that are able to be followed year-round. For small mammal and bird species however, it is often only viable to collect data for a limited, fixed, single-season period as a small battery size is necessary to avoid exceeding maximum percentage bodyweight threshold [27].

Trade-offs resulting from the incompatibility of low weight and long battery life may affect which individuals can be tracked [17,27] and may limit how much data can be collected. Movement patterns recorded may therefore be influenced by the parameters used when collecting tracking data [20], fix-acquisition bias [18,28], or method of analysis. The impact of variation in fix rate or duration of tracking period on resulting home ranges and habitat selection estimates is seldom explicitly considered or taken into account (but see [25,26]). However, it is important to ensure that data are collected at the most appropriate temporal scale in order to acquire data of a certain quality or quantity necessary to answer the questions posed.

Studies that report the implications of varying fix rate and duration of tracking period, often address these issues with simulated, rather than empirical data [20,29]. They also do so largely in the context of GPS fix failure [18], movement distance [30,31] or home range estimation often with VHF not GPS tags [25,29], rather than effects on estimates of habitat selection [18,19,25,26]. A small number of in-depth studies on estimating home range with conventional estimators, conclude that changing fix rate and duration of tracking can alter estimates of home range and consequently inferences about movement and behaviour [25,28,32], in part due to the effect these parameter changes have on the autocorrelation within the data [22]. Borger [25] identified tracking duration (number of days) as the key parameter influencing home range estimation, whilst Huck [20], Walter [33] and Byer [34] identified method of estimation as the most important factor for both home range size and proportion of habitats available. Stark *et al*. [21] found that movement-based home range estimation methods, such as the biased random bridge, handled missing GPS points of up to 75% of the total dataset better than conventional kernel density estimates and similarly Walter et al. [35] found that incorporating the temporal aspect of the data produced more reliable estimates.

Tracking data are inherently autocorrelated [24,36,37], although if fixes are taken infrequently enough so as to be longer than the autocorrelation timescale of the data, data can be considered independent [22,24]. The autocorrelation timescale is often interpreted as the time it takes for an animal to cross its home range [22,24]; the number of points that satisfy this assumption, equating to the number of home range crossings, are referred to as ‘effective sample size’ [22]. Not accounting for autocorrelation in the data can lead to bias and variation using traditional estimators, such as the KDE [24,37], whereas movement-based methods of range estimation such as the MKDE [38] and BBMM [39] do account for autocorrelation, but do not estimate ‘true’ home range, but rather the animal’s occurrence range [22,37]; a picture of where it has been, rather than what it necessarily needs long term. The recently introduced AKDE (‘Autocorrelated Kernel Density Estimator’) attempts to combine both the autocorrelation structure of the data and estimation of a traditional home range, estimating area used on the effective sample size which better represents the longer-term distribution of points [24].

The use and incorporation of autocorrelated data also relates to how the smoothing parameter of kernel home range analyses functions [36]. The smoothing parameter, or bandwidth, (commonly: ‘*h*’) influences the weight of each data point within the probability distribution function that creates the home range [40–42]. There is no consensus as to which bandwidth parameter to use, however it should aim to minimize variation in the size of the home range estimate between sampling frequencies and individuals [40,41] and should strike a balance between assigning an overly high influence to outer points, possibly resulting in disjointed home ranges where this may not make sense (under-smoothing) and averaging over outer points, thus disguising details of the foraging range (over-smoothing) [41,43].

Home ranges are also linked to the estimation of habitat selection by providing an individual measure of habitat availability [20,44]. As shape and size of the home range may depend on the configuration of the tracking schedule [32], as well as estimation method and bandwidth parameter [41], it can then influence the strength of habitat selection estimates [20,45]. However, the extent to which a decrease in fix rate and number of days tracked can directly affect these estimates, is largely unstudied. Few studies discuss the effects of tracking parameters on habitat selection and those that do mostly discuss habitat-related biases in fix collection [18,28], rather than decisions made regarding the fix rate and how this might influence duration and therefore the results obtained. Girard *et al*. [45] found, using empirical GPS data and simulated changes in fix rate with moose (*Alces alces*), that decreasing rate did not significantly alter habitat selection conclusions and that preferences for specific habitat types were clear even at low fix rates (e.g. 1 fix every 7 days). It should be noted however, that this research was conducted on a large, slow-moving mammal, with the ability to conduct a tracking study for multiple months, which is not the case for many small species such as bats and birds, which present a very different system to larger mammals [46]. The scale and timing of movement undertaken by large herbivores (deer, bison) [47] or carnivores could be orders of magnitude higher [48,49], causing positional autocorrelation to last for a number of days [50]. Not only that but small insectivores have higher energy requirements [51] and are exploiting a more spatially- and temporally-variable resource which will influence the time they spend moving and the configuration of their movements [52].

Given the increasingly widespread use of relatively cheap, miniature GPS units, it is pertinent that the influence of tracking parameters and data analysis methods are studied in the context of habitat use by species [53,54]. As such, this study is framed particularly in the context of the increased use of high-temporal resolution GPS units as opposed to VHF tags, on a small mobile central place forager. We concentrate particularly on how decisions made by researchers before deployment can influence analysis and results, as well as the use of a movement-based method of estimating home range, which has not been studied in the context of manipulation of these parameters.

### Study scenario

Our study focused on a migratory, insectivorous bird of conservation concern [55,56], the European nightjar *Caprimulgus europaeus* (hereafter referred to solely as ‘nightjar’). Nightjars are mostly single-brooded [57], nesting traditionally in dry heathland habitats with scrubland [58], mature trees and heather supporting good moth and beetle populations [55,59]. Their numbers fell significantly throughout Great Britain in the early part of the 20^th^ century due to afforestation, and loss of habitat [60]. Nationally, the population size has now stabilised [56], but threats such as climate change, urban development and agricultural intensification that remove both nesting and foraging resources, still continue [61,62]. Although nightjars are mobile and thought to be adaptable to land use change [63], they are also site-faithful [64] and there is little evidence in the literature to demonstrate their resilience to significant habitat transformation, particularly with smaller populations on atypical sites. Summer residency in northern Europe, including Great Britain, is short, lasting from May until September, with some females only arriving in mid-June [65]. This provides a limited window in which to track this species.

In this study, we tracked a number of individuals from a relatively stable breeding population of nightjars [66,67] on one of the more northerly breeding sites in Great Britain [68]. We aimed to determine the effect of fix rate and tracking duration from GPS data, on estimates of home range size and habitat selection and to assess the trade-off between fix rate and tracking duration in terms of the information gained about an animal’s area of use.

We had the following research questions:

1. How sensitive are estimates of home range size and shape to changes in fix rate and tracking duration?
2. How sensitive are estimates of habitat selection to any changes in tracking parameters and method of home range estimation and are the conclusions equivalent across all rates, durations and methods?

## Materials and methods

This work was carried out on the Humberhead Peatlands National Nature Reserve, South Yorkshire, which consists of Thorne Moors (53.636, -0.89682) and Hatfield Moors (53.545, -0.93493). The project was developed as part of an EU-funded LIFE+ project to monitor behavioural responses of European nightjars to habitat restoration. All fieldwork was subject to ethical approval through the University of York and was conducted with appropriate licences to capture and deploy tags onto birds through the British Trust for Ornithology.

The data consist of GPS fixes collected from 32 adult birds from 2015–2018, tracked over 6 or more days at two different rates. Birds were tracked from 21:00 to 05:00 hrs, but points spent at the roost in the first and last 30-minute periods (i.e. 21:00–21:30 and 04:30–05:00) were removed to avoid bias [69,70]. Data were collected using miniature nanofix GPS tags (Pathtrack, Otley, UK), at rates of 20 fixes per hour (n = 15), totalling 160 per 8-hour tracking session in 2015 and 2016, and 12 per hour (n = 17), totalling 96 per 8 hour tracking session in 2017 and 2018. This was equivalent to setting a 3-minute and 5-minute fix interval respectively, in the pre-programmed tag parameters. The tags weighed approximately 1.75g (equivalent to 3% or less of the bird’s bodyweight). In order to achieve such a small size, the battery and memory chip inside the GPS tags were necessarily small and their use requires a decision to be made on the trade-off made between fix rate and tracking duration. In 2017, the interval between fixes was increased from three to five minutes, thus decreasing the fix rate from 160 to 96 per session, in order to obtain an increased number of days of data, rather than collecting more fixes over fewer days.

### Habitat mapping

Habitat types across the study site were primarily mapped using supervised classification of Unmanned Aerial Vehicle (UAV) photographs within ArcMap (v. 10.5). We created a five-metre resolution habitat map, which was then updated in subsequent breeding seasons using hand-held GPS units on site, to incorporate annual habitat management activities. Thirteen habitats were classified, taking into account both vegetation type and structure, both thought to be important to nightjars.

### GPS data processing

The data were processed and analysed in R (v.3.5.1). In order to explore the effect of fix rate on estimates of home range and habitat selection, the original data were subsampled. Firstly, fix rate was halved according to the initial rate (i.e. 6 or 10 fixes per hour, totalling 48 or 80 fixes per 8 hour session, equivalent to a 6- or 10-minute fix interval); secondly the data were subsampled to give a rate of four fixes per hour (i.e. 32 per session or a fix interval of 15 minutes). To investigate the effect of tracking duration on home range and habitat selection, the full datasets for each bird were subsampled into the first 3- and 6-day periods.

These data were then used to estimate individual home ranges using both the Biased Random Bridge method for movement-based kernel density estimation (MKDE) [71,72] and static kernel density estimation (KDE), using package ‘adehabitatHR’ [73]. These represent one of the most commonly used methods of range estimation and a more recently developed occurrence estimator, or movement-based home range, that explicitly uses the connections between tracking data fixes to identify heavily-used areas and corridors [38]. For the MKDE, specific movement information gathered from the tracking data was used to parameterise a more descriptive, movement-based home range [74], compared with the KDE method. Each GPS fix is associated with a timestamp (date and time combined), meaning the exact time between fixes is calculated. Specific calculations include: a diffusion parameter comprised of the maximum time permitted between fixes (‘*Tmax*’; here, we have used 3 x fix frequency, i.e. either 9 or 15 minutes [38]) and the minimum distance that represents movement (10 metres) [38]. The inclusion of the ‘Tmax’ value therefore excludes the 16 hour gap present in the schedule that occurs while the units are switched off during the day. As a central place forager, the nightjar is constrained to its nest or roost during the day when it is unable to feed, thus, the inclusion of this area would likely bias the home range unfairly downwards, as with seabirds constained to nesting on land [75]. Constructive home ranges for such constrained foragers is difficult, but this decision likens the nightjar MKDE to an ‘active’ home range as in [76]. These parameter values are used in conjunction with a variable smoothing parameter applied to different parts of the track, which is calculated from values chosen by the user. These values are ‘*hmin*’, a value in the units of the GPS locations, chosen to balance the GPS-related error and the mean distance moved between points (here, 60 metres); becomes ‘*hmax’* at the interpolated point furthest from two known locations[38,72,77]); ‘*Tmax’* and the grid size (here, this was the underlying 5 x 5 metre habitat map). The smoothing parameter used within the KDE analysis was ‘*href*’, also referred to as the reference bandwidth [41,78], which is estimated using the standard deviation of the x and y coordinates[78]. This was used in preference to the ‘LSCV’ method, which tends to undersmooth [42] and may less accurately account for the possible distance travelled between points, especially by such a mobile bird that can cross its home range very quickly [24]. We used the variable smoothing parameter and *href* throughout the analysis for all full- and subdivided samples, to avoid adding variance and bias into the study related to this parameter. We anticipated that as the MKDE has been found to cope better with missing points, it should also maintain an accurate representation of animal space use even with a decreased fix rate [21]. Home range sizes using both MKDE and KDE were calculated only for the 95% level as this is the most commonly used level in the literature.

Habitat availability within individual 95% home range estimates was identified using the ‘over’ function in sp [79]. Home range habitat availability was combined with used points, identified using the ‘join’ function in adehabitatMA [73], excluding points outside of the home range boundary, to estimate Manly Selection Ratios [80]. These were estimated using the ‘widesIII’ function in adehabitatHS [73], where use and availability differ between individual animals and as such, a selection ratio is produced for each habitat type along with an overall selectivity measure of an individual bird across all habitats [73,80]. Here we use the latter, termed within the adehabitatHS package as Khi2Lj, that incorporates all single-habitat selection ratios within each individual, into a combined measure of habitat selection (from here-on we will refer to this as the selection statistic). These selection values are a special case of the more-commonly used Resource Selection Function (RSF; [3,80]) and estimating habitat selection in this way provides a simple, easily-interpreted statistic, that makes better use of a single variable containing multiple categories, such as the habitat type variable in our study [80,81].

### Autocorrelation assessment

To further understand the results from the MKDE and KDE home range estimation, it is important that the underlying structure of the data is assessed [37]. We visualised data from all individuals, using variograms and correlograms in package ‘ctmm’ [23], to gather information relevant to home range estimation such as positional- and temporal-time-to-independence. We then ran AKDE home range estimation analyses, which incorporate an underlying movement model into the estimation of a ‘true’ home range [24], using Ornstein-Uhlenbeck foraging (OUF) model-estimated variance and bandwidth parameters model [23,82] that brings in both positional and velocity autocorrelation. The values produced for these were then compared to those produced from the KDE and MKDE to observe any differences cause explicitly by the autocorrelation structure of the data.

Variograms displayed immediately strong autocorrelation, followed by a rapid but individually-variable asymptote (S1 Appendix). The data possessed strong positional and velocity autocorrelation within the first 30 minutes of tracking, which equates to 10–12 or 6–8 fixes at the two sample rates (160 or 96 per day), demonstrating that to achieve true independence the data would need to be subsampled to a 30 minute fix interval (approximately 16 per day), far less frequently than currently taken. However, the relationship between the size of the area traversed by the individuals, meant that effective sample size was still high. This highlights that although there is autocorrelation in such frequently acquired data, for a central place forager holding a small home range relative to the tracking duration, this is not as significant as it would be for an animal traversing a larger area, relative to the fix rate [22,24]. This resulted in no significant difference between KDE and AKDE home range sizes (ANOVA, F_2,536_: 19.93, p < 0.0001; Tukey post-hoc tests: MKDE:KDE p < 0.0001; MKDE:AKDE p < 0.0001; KDE:AKDE p = 0.57; S4 Appendix).

Consequently, we have analysed the data for habitat selection with the KDE and MKDE, to demonstrate the use of both a range and an occurrence estimator with data that is initially strongly autocorrelated but asymptotes quickly, relative to the total length of tracking.

### Modelling

Estimates of home range size and habitat selection, for all home range estimation methods, were then brought into linear mixed effects models using the ‘lmer’ function in lme4 [83]. These mixed effects models were able to identify the influence of both spatial and temporal variables using fixed effects, as well as identifying individual variation in these variables, using random effects. Methods such as this to deal with individual variance, i.e. mixed-effects models, are being used more widely [84,85] and prior exploratory analysis in this study showed clear influence of the individual bird on the strength of the response to change in the tracking duration and fix rate. Both response variables, home range size and selection statistic, for both methods were log transformed for normality [25,53].

Four separate models were created (Table 1). Variables were subject to prior exploratory analysis related to *a priori* hypotheses. Sex of the bird did not have an influence on the result and was thus not included. Two models were run for the two different home range estimation methods, in order to test the sensitivity of the home range estimates to variation in tracking parameters, followed by two habitat selection models, to test the sensitivity of the habitat selection estimates to variation to the same tracking parameters.

**Table 1:** Outline of the four linear mixed models used in analysis. Response variable is modelled against the corresponding fixed and random effects listed in each row.

Response variable	Fixed effects	Random effects
1. MKDE/KDE/AKDE Home Range size (hectares)	Number of days + Fix rate + Number of fixes + Year + Site + Dominant habitat	1. Individual (intercept) / Days (slope) 2. Week number
2. Habitat selection statistic (derived from MKDE Home Range)	Number of days + Fix rate + Number of fixes+ Year + Site	1. Individual (intercept) / Days (slope) 2. Week number

Fixed effects in all starting models were:

1. Tracking Duration: number of days, ranging from 3 to 17.
2. Fix Rate: expressed as the number of fixes per session; one of 32, 48, 80, 96 or 160 (corresponding to 4, 6, 10, 12 or 16 fixes per hour).
3. Number of fixes; the total number of fixes in a bird’s full, or subset dataset.
4. Year; either 2015, 2016, 2017, 2018.
5. Site; Hatfield or Thorne.

Dominant habitat, representing the habitat type within a bird’s home range (derived from the MKDE or KDE polygon and overlaid on a five-metre resolution habitat raster) with the highest number of pixels (i.e. largest availability) was included as a fixed-effect only in the home range models. Random effects to account for variation in the coefficient values, were the same for all models and included Individual and tracking duration as the random intercept and slope respectively (Table 1). Including tracking duration as both a fixed effect and a random slope [86] aimed to improve the fit of the model by recognising individual variation in response to changing tracking duration, something that was uncovered during the prior exploratory analysis. Week of the breeding season in which the bird was tracked was also included as a random effect.

To directly compare the impact of the parameters on the data originally collected at two different fix rates, we subsampled all data to a 15-minute interval. We again ran four models with the same starting dependent variables of home range size and habitat selection statistics, which did not include fix rate as a fixed effect, but did include tracking duration, temperature, year, habitat and site, to attempt to unpick underlying variation. AICc (AIC corrected for small sample size) was used to judge the most appropriate model for all analyses. We followed a stepwise selection procedure, whereby dropping single terms from the model resulted either in a decrease or increase in AICc value. The final model was determined when no further decrease could be achieved by removing single terms. Single terms were added back into the final model, in a random order and a secondary model selection procedure was employed using MuMin (v. 1.42.1; [87]) in R, to check the validity of the reduced model. Fit of the final models was assessed through normality of the residuals using the plot function in package ‘lme4’ (v. 1.1–17) and by simulating residuals and testing for uniformity in package ‘DHARMa’ (v. 0.2.0; [88]). Where model selection did not achieve delta AIC > 2, i.e. there was no ‘best’ model, we used the ‘model.avg’ function in MuMin and produced model-averaged parameters. Final model coefficients for both fixed and random effects are presented in the results. As response variables were log-transformed, the values are presented accordingly as percentage increase in y, with a 1-unit increase in x.

## Results

### Home range information

Across the whole dataset of 32 birds, the mean (+/- SD) home range sizes were 204.04 ha (+/- 229.42; MKDE) and 115.1 ha (+/- 153.62; KDE; Table 2; S2 Appendix). All estimators varied between and within fix rate and day subsets; MKDE range sizes were at their highest at the lowest fix rate of 32 fixes per day (342.88 ha +/- 327.61), whereas KDE range sizes were largest in the 5-minute fix interval category (125.25 ha +/- 182.61; see S2 Appendix). Mean values for the shortest tracking duration subset of 3 days were 138.57 ha (+/- 167.11) for MKDE; 109.84 ha (+/- 184.89) for KDE (Table 2). Large standard deviations represent high individual variation, addressed in much more detail in the following sections (and see S2 Appendix).

**Table 2:** Mean values (+/- S.D.) for MKDE and KDE estimated home range sizes (hectares) for each fix rate subset and two shorter duration subsets within the dataset (mean value across all subsets per year). Sample sizes vary between subsets; 16 and 10 fixes per hour, n = 9; 12 and 6 fixes per hour, n = 23; 4 fixes per hour, n = 32; 3 days, n = 64; 6 days, n = 32.

	*At a fix rate of*:						*At a subset of*:	
	16/ hour	12/ hour	10/ hour	6/ hour	4/ hour	All	3 days	6 days
**Mean MKDE (ha)**	94.74	179.87	158.04	260.89	342.88	204.04	138.57	163.42
**(+/- S.D.)**	92.13	187.94	195.18	235.4	327.61	229.42	167.11	162.98
**Mean KDE (ha)**	80.81	125.36	104.5	118.96	119.17	115.1	109.84	91.53
**(+/- S.D.)**	91.49	182.61	140.76	117.54	133.08	153.62	184.89	118.92

### Modelling results

#### Home range

**Fig 1:**
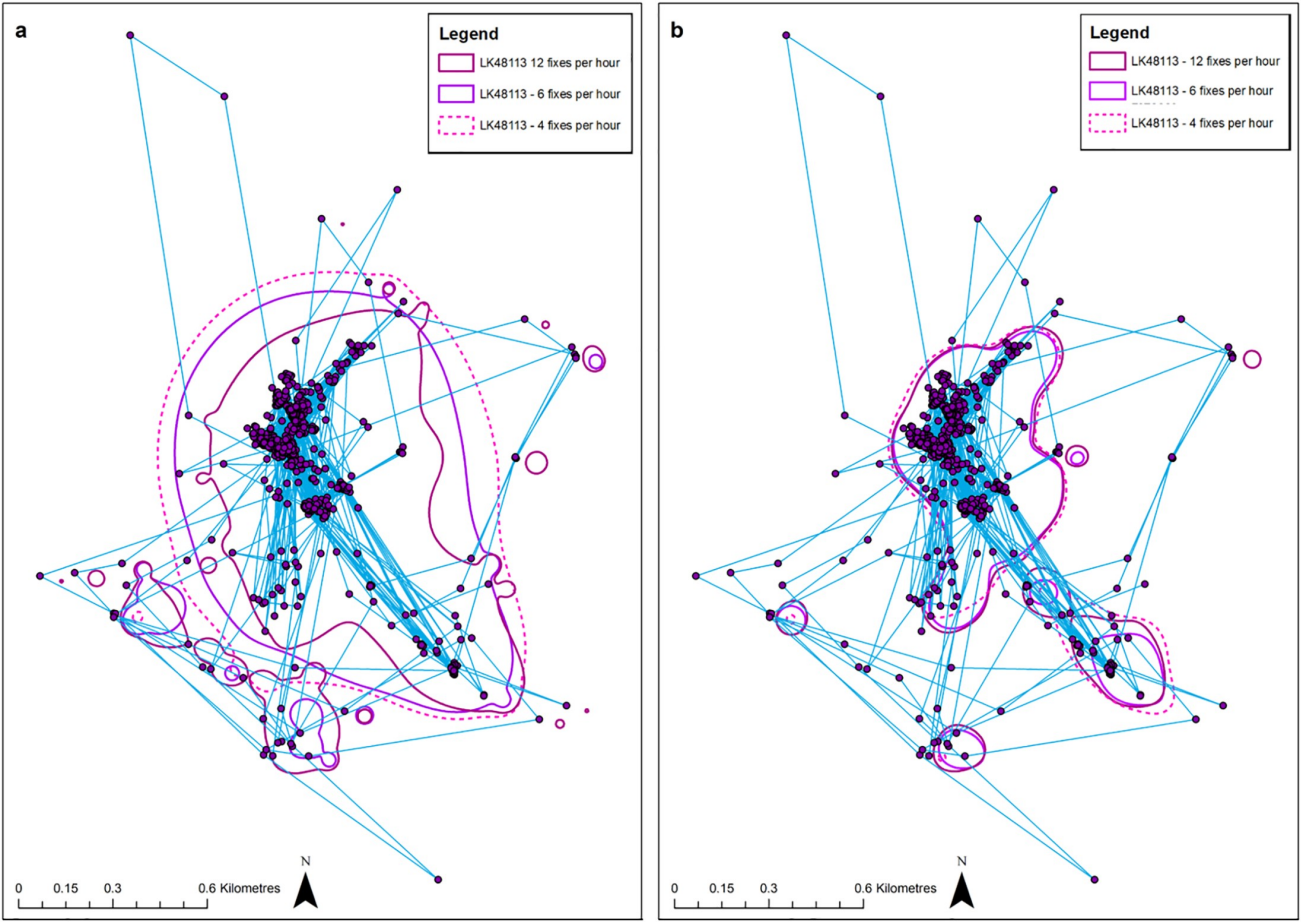
Example of MKDE and KDE home ranges calculated at three different fix rates. Estimates of MKDE (A) and KDE (B) were calculated at the standard rate (12 fixes per hour or 96 per 8-hour tracking session, equivalent to a 5 minute fix interval) and the two subsampled rates of 6 fixes per hour (48 per session, equivalent to a 10 minute fix interval) and 4 per hour (32 fixes per session, equivalent to a 15-minute fix interval), as identified in the key. GPS fixes outside of the home range polygons have been excluded from the habitat selection calculations.

To test the influence of multiple tracking parameters on estimates of home range size, we ran three models with MKDE and KDE sizes as the dependent variable. For both estimators, tracking parameters were influential (Fig 1, Table 3). MKDE home range size was most strongly influenced by fix rate and tracking duration (Table A in S3 Appendix). Dominant habitat type within the individual’s area was also influential, whilst number of fixes, site and temperature had a negligible influence and were removed. The final model indicates that every one-unit decrease in the fix rate results in a -0.59% change in home range size, i.e. the lower the fix rate, the fewer fixes collected per day and the larger the home range (Table 4).

**Table 3:** Final model coefficient estimates and random effect variance parameters for each of the four models run to explore factors affecting home range and habitat selection. 95% confidence intervals are presented in brackets, following fixed effect coefficients. Standard deviation is presented in brackets following random effect coefficients.

	*Coefficient estimates*			
*Predictors*	MKDE Home Range	KDE Home Range	MKDE Habitat Selection	KDE Habitat Selection
Intercept	7.049	4.234	4.65	4.179
	(5.871–8.228)	(2.56–5.909)	(4.187–5.113)	(3.763–4.594)
Fix Rate	-0.006		0.001	
	(-0.007–-0.004)		(-0.0005–0.006)	
Tracking Duration	0.039	0.034	0.048	0.03
	(0.007–0.071)	(0.002–0.066)	(0.009–0.087)	(0.012–0.049)
Number of fixes			0.001	0.001
			(0.0006–0.002)	(0.001–0.002)
Site		0.184	-0.408	-0.245
		(-0.256–1.165)	(-0.814–-0.002)	(-0.941–0.036)
Dominant habitat	+	+		
*Random effects*				
*Intercept/Individual*	0.343	1.234–1.244	0.261–0.269	0.62–0.752
*(*+/- SD)	(+/- 0.585)	(+/- 1.111–1.115)	(+/- 0.511–0.518)	(+/- 0.788–0.862)
*Days/Individual*	0.004	0.005	0.0004–0.0005	0.001
(+/- SD)	(+/- 0.062)	(+/- 0.072)	(+/- 0.019–0.022)	(+/- 0.028–0.031)
*Intercept/Date*	0	0.00–0.098	0.013–0.03	0.04–0.089
(+/- SD)	(+/- 0.000)	(+/- 0.314)	(+/- 0.112–0.174)	(+/- 0.20–0.299)
*Sigma (Resid*. *var*.*)*	0.091	0.101	0.118–0.121	0.09
(+/- SD)	(+/- 0.302)	(+/- 0.318)	(+/- 0.343–0.348)	(+/- 0.30)

**Table 4:** Influence of tracking parameters on MKDE and KDE home range and habitat selection. For every one-unit increase in the variables in the left-hand column, there was a change in the corresponding home range or habitat selection estimate, given in percentage increase or decrease.

	MKDE		KDE	
*Increase per unit in*:	Home Range	Selection statistic	Home Range	Selection statistic
**Fix Rate (Fixes per session)**	**↑** 0.59%	**↑** 0.3%	^1^NA	NA
**Tracking Duration (Days)**	**↑** 4%	**↑** 4.92%	**↑** 3.46%	**↑** 3.01%
**Number of fixes**	NA	**↑** 0.1%	NA	**↑** 0.1%

^1^NA where variable did not appear in final model.

**Fig 2:**
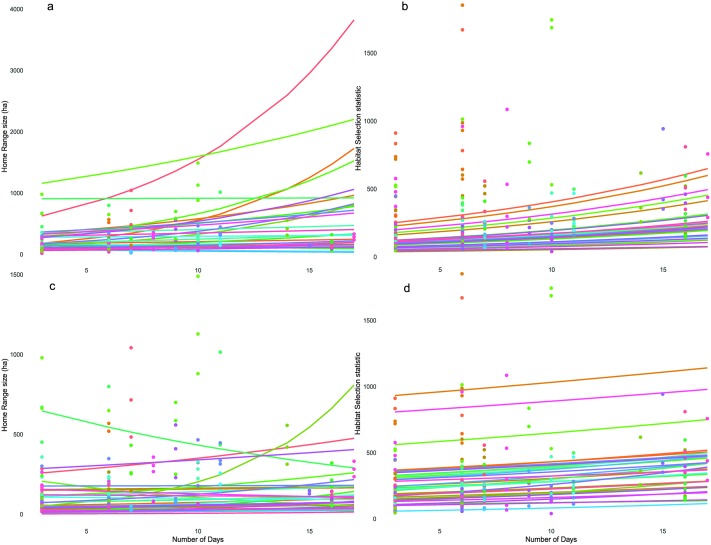
Outputs from the linear mixed models showing variation in individual response to altered tracking duration in home range size or habitat selection, for both home range estimation methods. Panels a and b display results of the home range and habitat selection analysis using the MKDE; panels c and d display results using the KDE. Predictive regression lines are displayed for each individual (n = 33). NB: different y-axis scales on each plot.

A one-day increase in tracking duration equated to a 4% increase in home range (Table 4, Fig 2). This final model containing just Fix rate, tracking duration and habitat held most of the model weight (0.63; Table A in S3 Appendix). Individual as a random effect accounted for the majority of the variation in MKDE home range size (Table 3). Number of days (included as a random slope) explained only a small amount of extra variation (0.004; Fig 2).

Tracking duration and dominant habitat were the most influential parameters when estimating KDE home range size (Table B in S3 Appendix). Also, contradictory to the estimates from the MKDE, fix rate had minimal impact (Fig 1). An increase of one day resulted in a 3.46% increase in the KDE home range size (Table 4, Fig 2). However, several of the reduced models held similar AICc values, resulting in model-averaged parameters from the best 2 models (Table 3; Table B in S3 Appendix), the second of which also included site. These two models combined held an Akaike weight of 0.7 (Table B in S3 Appendix). Variance attributed to individuals was higher than for MKDE home range (Table 3); further to individual random variation, tracking duration also provided some explanation of the variance along with residual variation.

#### Habitat selection

To assess the sensitivity of habitat selection estimates to changes in tracking parameters, variation in the estimated habitat selection statistic [80], derived from both home range estimators was modelled against tracking parameters, and weather and temporal covariates. For the MKDE-derived habitat selection, fix rate, the number of fixes, tracking duration and site (Table 3; Table C in S3 Appendix) were significant, but the top model was within ΔAIC 2 of the second ranked model, therefore these have been averaged. The removal of the total number of fixes resulted in an increase in AICc of >20 and its inclusion provided the most weight to the final model selection table (Table C in S3 Appendix). An increase of one fix resulted in a 0.1% increase in selection strength (Table 4), which although small was significant. An increase in fix rate by one unit resulted in an increase of 0.3% in the selection estimate, whilst an increase in tracking duration caused an increase in the selection estimate of 4.9% per day. Unlike the home range models, site on which the birds were tracked was heavily influential, with a 98% decrease in selectivity from Hatfield Moor to Thorne Moor (Table 3). Individual differences accounted for a considerable amount of the random variation, along with tracking duration (Table 3). Date-related variation was negligible, but higher residual variation was present (Table 3).

For the KDE-derived selection statistic the number of fixes had the most weight, and increased AICc by >100 if dropped from the model (Table D in S3 Appendix). Tracking duration was also important, with site less important but still relevant to the model. As with MKDE-derived habitat selection, the top model was within ΔAIC 2 of the second best, so these were model averaged. An increase of one fix, resulted in a 0.1% increase in KDE-derived selection (Table 4).

Likewise, an increase in tracking duration of one day, caused a 3% increase in SR (Table 4). Again, site influence was clear, although varied between individuals, with a decrease in selectivity when moving from Hatfield Moor to Thorne Moor (Table 3). The magnitude of the individual variation was stronger than when estimating MKDE selection (Table 3). Both the intercept and slope of the home range and habitat selection models vary between individuals (Fig 2). Home range both increases and decreases with an increased tracking duration, depending on the individual. The relationship is clearer for habitat selection, where an increased tracking duration leads to an increased habitat selection statistic, indicating higher selection strength (Fig 2).

#### Direct data comparison

We carried out additional analysis of the tracking data at a 15-minute fix interval where direct comparison among years was possible, in an attempt to understand if changes in fix rate over the course of the study might have masked other changes. For each dependent variable, a different set of parameters were most influential (Table A in S4 Appendix). Only within the MKDE home range analysis was there a clear effect of year, with home range size increasing from 2015 linearly through to 2018 but decreasing with temperature (Table 5). In comparison, KDE home range size was influenced most strongly by the tracking duration and number of fixes. Neither habitat selection model displayed an effect of year; habitat selection derived from the MKDE home range was influenced by tracking duration, but also temperature, whereas that derived from KDE home range was only influenced by site (Table 5; Table A in S4 Appendix).

**Table 5:** Model coefficients from four models testing the effects of tracking-parameter-related, temporal and weather covariates. Data were subsampled to a 15 minute fix interval (32 fixes per day, n = 32). Models tested the influence of parameters on MKDE and KDE home range and habitat selection estimates. 95% Confidence intervals in brackets.

	*Coefficient estimates from models testing effects on*:			
*Predictors*	MKDE Home Range	KDE Home Range	MKDE Habitat Selection	KDE Habitat Selection
Intercept	7.499	3.75	6.443	4.887
	(4.36–11.363)	(2.688–4.865)	(4.666–8.211)	(4.581–5.192)
Tracking Duration		0.249	0.068	
		(-0.077–0.586)	(-0.005–0.141)	
Number of fixes		-0.007		
		(-0.019–0.004)		
Site				-0.385
				(-0.864–0.095)
Year	+			
Temperature	-0.183		-0.112	
	(-0.346–0.01)		(-0.219–-0.004)	
**Random effects**				
*Week number*	0.033	0.059	0.089	0
(+/- S.D.)	(+/- 0.182)	(+/- 0.526)	(+/- 0.299)	(+/- 0.000)
*Sigma (Residual var)*	0.642	0.99	0.315	0.435
(+/- S.D.)	(+/- 0.801)	(+/- 0.995)	(+/- 0.562)	(+/- 0.66)

## Discussion

Manipulation of tracking parameters influenced all aspects of our study results in some form. All the factors presented here have relevance for researchers looking to plan their own tracking study and should at least be considered, as they may mask other elements. All parameter values should be reported to allow for full understanding of the results. We have provided information on the magnitude of the change in home range and habitat selection where possible, to aid understanding of the strength of the relationships between variables should researchers need to make this trade-off when studying a small species for which ‘unlimited’ tracking is not possible. Below we discuss these factors in the context of our original research questions and in the context of movement research overall.

### How sensitive are estimates of home range size and shape to changes in fix rate and tracking duration?

Both methods of home range estimation were sensitive to tracking duration, but only the MKDE was sensitive to fix rate. The influence of fix rate on MKDE, is a reflection of the autocorrelation assumptions within the method and the underlying structure of the data [22,37,72]. For the MKDE, the density of, and the space between, consecutive points is weighted, which means that if fix rate were decreased in order to extend tracking duration, this would increase the area in which there is a probability of finding the animal (creating more uncertainty), producing a larger MKDE.

Sensitivity to tracking duration of both methods identifies this as a key variable. A longer tracking duration means that extra information is gained, producing a larger sample size [22]. For species that have previously only been tracked for short periods, the information gained from extra days of tracking could be very valuable, because what animals do for a few days is not necessarily representative of what they do longer term. Where the relationship between home range crossing time and frequency of fixes gives rise to strongly autocorrelated data (i.e. crossing time exceeds the interval between fixes), longer tracking enables the effective sample size to increase, making the results more interpretable [16]. We identified strong bias in the estimation of home range size if data are collected for only a few days for both estimators, due to a substantial amount of both between- and within-individual variation in foraging locations. Within- individual variation in movement behaviours is also identified by Fleming and Calabrese [22] as a constraint to standardisation across different tracking durations. Therefore we recommend that researchers acquire tracking data over a longer duration not only to provide a more balanced understanding of where the animal is going in the presence of strong individual variation, but to increase effective sample size [24]. This contrasts with recent information from a study of the large mammal literature by Hofman *et al*. [54], who recommend more regular tracking than is thought necessary in order to counteract issues with retained ephemeris data and fix acquisition (see also [89]).

In our study, the MKDE provided an accurate representation of used areas and is therefore suitable for habitat selection and resource use analyses, particularly when observing year to year changes, due to its position as an occurrence rather than a range estimator [37]. However, the influence of tracking parameters on this method means this might not be true for larger, slower moving animals, such as deer [35,90], compared with small, mobile species such as the nightjar. The spatial and temporal scales over which species of different sizes and traits operate, will influence appropriate data collection schedule (and thus autocorrelation). Large herbivores such as deer or moose [91] track resources that may only vary over a weekly- or monthly timescale, and therefore may only necessitate daily fixes. Nightjars and other small, insectivorous aerial foragers [92] track mobile resources that may vary on shorter timescales related to daily weather conditions and small-scale spatial changes in temperature [62,93,94], the effects of which may be amplified by habitat type and structure in their home range, differing by metres rather than kilometres [95,96]. Nightjars are visual predators that feed on-the-wing, making the connections between points and not just stationary locations, more important. Therefore, to quantify changes on this scale requires shorter tracking intervals. The increase in MKDE home range size with a longer tracking duration, along with strong individual variation signals the need for tracking data to be analysed with a method appropriate for its structure. Consequently, we suggest that researchers undertaking any movement-based kernel analysis, to do so at a standardised rate across individuals, or to use analysis methods that incorporate varying autocorrelation structures, such as the AKDE.

### How sensitive are estimates of habitat selection to any changes in the tracking parameters and method of home range estimation and are the conclusions equivalent across all rates, durations and methods?

The strong influence of number of fixes for both MKDE and KDE-derived habitat selection is partly explained by Manly selection statistic calculation methods, as this method considers how many points are selected in each different habitat and compares this to the respective relative availabilities, and collates this information over all habitat types used and available per individual [80]. Each extra fix collected adds weight to the use of each habitat, compared to its availability, and the relationship becomes stronger if availability does not change. Whilst we recognise the limitations of the selection ratio method, we believe it is an intuitive method with which to observe habitat selection and preferences of animals when faced with a simple habitat-type metric, that would struggle to be modelled in a linear format [3,80].

Fix rate and tracking duration influenced habitat selection estimates derived from the MKDE and KDE home ranges respectively. Decreasing the fix rate could decrease the level of habitat selection as calculated with the MKDE home range. Firstly, because there are simply fewer fixes in total, but also due to the longer interval between fixes, the autocorrelation has reduced and the animal is potentially less likely to be in the same place, particularly for a very mobile aerial feeder such as the nightjar, which can cross its home range in less than the time between consecutive fixes. If the decrease in fix rate results in an increased tracking duration due to battery life and/or memory space, a similar level of selectivity may be reached during the extra tracking time, particularly if individuals are consistent in their foraging. We achieved the same number of fixes over a 10-day tracking period at a lower fix rate, as we did over only 6 days at the original, higher rate, providing us with an almost 50% increase in the number of days of data, with a reduction of only 8 fixes per hour, or 64 per tracking session.

It is also important to note that habitat selection estimates from both the MKDE and KDE were sensitive to the site studied, which concurs with Borger *et al*.[19] and Byer [34]. This suggests that selection estimates could be sensitive to habitat configuration as well as method. Bearing in mind that home range size dictates the individual availability of habitat to calculate the habitat selection, change in the home range size with method could result in inclusion of different habitat types, ultimately influencing the resulting habitat selection ratios. Animals could appear to be much more selective if they use habitats that are sparsely distributed, necessitating some commuting behaviour across large areas of unsuitable habitat, which if modelled with the MKDE, rather than the KDE, may lead to much larger, contiguous areas of available, but unused, habitat being included.

Strong individual variation in the habitat selection estimates were particularly related to tracking duration. Week number only explained some of the variation in habitat selection estimates, not those of home range, which is likely to reflect changes in food availability and weather conditions. Ultimately in this study, although the selection estimate changed with number of fixes, the primary conclusions (i.e. the most selected habitat) did not change, (in accordance with Girard [45]), although occasionally the precise order in which habitats were selected did.

Models run with subsampled data, therefore making the results directly comparable across the individuals in the population, show that external factors (temperature, site, year) not dictated by the tracking parameters are influential. This clarifies the need to track individuals at the same rate and for the same duration, to allow the effects of these parameters to be more evident. In particular, variation in home range estimates and habitat selection due to site and year, could reflect differences in vegetation type and structure and may indicate the potential for there to be underlying differences in fitness, survival or breeding success [97]. These models also highlight the difference between the range and occurrence estimators; the latter (MKDE) uses movement parameters within the data and here has highlighted a decrease in home range with temperature and year, external influences not picked up by the range estimator (KDE).

## Conclusions

Fix rate and tracking duration acquired from miniature GPS units influenced the results of our tracking and habitat selection study, where the size of the species restricted the type of tag and consequently a trade-off was made between fix rate and tracking duration. We concur with recent literature on autocorrelation; changing fix rate alters data structure. We recommend that data are analysed in accordance with autocorrelation structure and the ecology of the species; an understanding of scale in temporal and spatial movement is necessary to achieve a high effective sample size. For a small, mobile central place forager such as the nightjar, which can travel rapidly across its home range and is exploiting localised, temporary resources, it is important to maintain the data collection at a sufficient schedule so to balance small-spatial scale movements with longer-term changes in prey distribution that can provide information about their needs for productivity and survival. Overall, we recommend tracking animals for as long as possible, to reduce the skew and bias that can arise from individual variation in movement patterns, so as not to make conservation recommendations based on potentially unusual behaviour. The overall conclusions from our habitat selection analyses however, did not change, despite the estimate of habitat selection strength changing by some magnitude. Therefore, for species where the main concern is to identify priority habitat type for conservation, more infrequent fixes over a longer time will suffice.

## Supporting information

S1 Appendix Examination of autocorrelation of nightjar GPS data. Examination of autocorrelation structure including time to independence using variogram and correlogram tools within ‘ctmm’[23].
(DOCX)

S2 Appendix Data compiled from analysis of GPS tracks of European nightjars. Data includes home range and habitat selection estimates from the KDE and MKDE methods, as well as home range calculated using the AKDE.
(CSV)

S3 Appendix Model selection tables for linear mixed models produced to test effects of tracking parameters on two home range estimators and their respective habitat selection estimates. Models reduced by AICc and df; where models were within delta AIC2 models were averaged.
(DOCX)

S4 Appendix Model selection tables for linear models produced from all subsampled data. Models produced to test for effects of tracking duration and other covariates across all individuals sampled at the same rate of 4 fixes per hour (n = 32).
(DOCX)
