# Supplementary material for: The trade-off between fix rate and tracking duration on estimates of home range size and habitat selection for small vertebrates
Source: PLoS One. 2019 Jul 10;14(7):e0219357. doi: 10.1371/journal.pone.0219357 (PMC6619758; doi:10.1371/journal.pone.0219357)
Supplement: S1 Appendix — Examination of autocorrelation structure including time to independence using variogram and correlogram tools within ‘ctmm’[23]. (DOCX) [file pone.0219357.s001.docx]

**S1 Appendix**

**Examination of autocorrelation of nightjar GPS data.** Examination of autocorrelation structure including time to independence using variogram and correlogram tools within ‘ctmm’[1].

**Figure A: Examples of autocorrelation variograms produced from nightjar GPS data.** Variograms show the Semi-variance function (SVF) plotted against the time-lag of the GPS data and typically asymptote where autocorrelation declines. N = 3 different individuals.

**Figure B: Examples of correlograms of autocorrelation structure produced from nightjar GPS data.** Correlograms show autocorrelation plotted against the time-lag of the data. N = 3 individuals; same individuals used as above, for direct comparison.

**Table A: ‘Autocorrelated Kernel Density Estimators’ (AKDE) home range sizes in hectares, with standard deviation.** Samples sizes vary between subsets. For 16 and 10 fixes per hour, n=9; for 12 and 6 fixes per hour, n=23; for 4 fixes per hour, n =32. For the tracking duration subsets; 3 days, n =64; 6 days, n = 32.

**Figure C: Size variation in hectares, within and between MKDE, KDE and AKDE home range estimators.** MKDE_95 = Movement based kernel density estimate (95% level); KDE_95 = conventional kernel density estimate (95% level); AKDE_HR = Autocorrelated kernel density estimate (95% level). Boxes display means, 1^st^ and 3^rd^ interquartile ranges; filled points = outliers.

**Figure D: Comparison of home range sizes estimated by KDE and AKDE.** Direct comparison of estimated size of individual nightjar home ranges, at each fix rate and tracking duration subset, as well as the full dataset. Home ranges calculated at the 95% level.
