# Supplementary material for: The trade-off between fix rate and tracking duration on estimates of home range size and habitat selection for small vertebrates
Source: PLoS One. 2019 Jul 10;14(7):e0219357. doi: 10.1371/journal.pone.0219357 (PMC6619758; doi:10.1371/journal.pone.0219357)
Supplement: S3 Appendix — Models reduced by AICc and df; where models were within delta AIC2 models were averaged. (DOCX) [file pone.0219357.s003.docx]

**S3 Appendix**

**Model selection tables for linear mixed models produced to test effects of tracking parameters on two home range estimators and their respective habitat selection estimates.** Models reduced by AICc and df; where models were within delta AIC2 models were averaged.

**Table A: Model table for MKDE Home range as the response variable**. ‘logLik.’ = Log likelihood; ‘dev’ = model deviance; Wi = Akaike weights. Model with lowest AICc highlighted in bold.

**Table B: Model table for KDE home range as the response variable.** ‘logLik.’ = Log likelihood; ‘dev.’ = model deviance. Wi = Akaike weights. Model(s) with lowest AICc value in bold.

**Table C: Model table for MKDE-derived Selection Ratio as the response variable.** ‘logLik.’ = Log likelihood; ‘dev.’ = model deviance. W_i_ = Akaike weights. Model with lowest AICc in bold.

**Table D: Model selection table for KDE-derived Selection Ratio as the response variable**. ‘logLik.’ = Log likelihood; ‘dev.’ = model deviance. W_i_ = Akaike weights. Model with lowest AICc in bold.
