# Supplementary material for: The trade-off between fix rate and tracking duration on estimates of home range size and habitat selection for small vertebrates
Source: PLoS One. 2019 Jul 10;14(7):e0219357. doi: 10.1371/journal.pone.0219357 (PMC6619758; doi:10.1371/journal.pone.0219357)
Supplement: S4 Appendix — Models produced to test for effects of tracking duration and other covariates across all individuals sampled at the same rate of 4 fixes per hour (n = 32). (DOCX) [file pone.0219357.s004.docx]

**S4 Appendix**

**Model selection tables for linear models produced from all subsampled data.** Models produced to test for effects of tracking duration and other covariates across all individuals sampled at the same rate of 4 fixes per hour (n = 32).

**Table A: Model selection table for response all response variables analysed at a subsampled rate of 32 fixes per hour for direct comparison.** Models reduced by AICc and df. ‘logLik.’ = Log likelihood. Model with lowest AICc in **bold**. Where models are within Delta 2 AIC of each other, model averaged coefficient values are obtained and reported in the text.
